# Use of 3D-computed tomography angiography for planning the surgical removal of pineal region meningiomas using Poppen's approach: a report of ten cases and a literature review

**DOI:** 10.1186/1477-7819-9-64

**Published:** 2011-06-15

**Authors:** Yunqian Li, Gang Zhao, Honglei Wang, Wanan Zhu, Limei Qu, Ye Li, Jinlu Yu

**Affiliations:** 1 Department of Neurosurgery, The First Bethune Hospital of Jilin University, 71 Xinmin Avenue, Changchun 130021, China; 2 Department of Radiology, The First Bethune Hospital of Jilin University, 71 Xinmin Avenue, Changchun 130021, China; 3 Department of Pathology, The First Bethune Hospital of Jilin University, 71 Xinmin Avenue, Changchun 130021, China

**Keywords:** Pineal region, meningiomas, 3D-CTA, Poppen's approach

## Abstract

**Background:**

There are several treatment approaches for pineal region meningiomas, such as Poppen's approach, Krause's approach and combinations of the two approaches. We present our experience with the use of 3D-computed tomography angiography for planning the surgical removal of pineal region meningiomas using a suboccipital transtentorial approach (Poppen's approach) and evaluate the role of Poppen's approach.

**Methods:**

During the period from January 2005 to June 2010, ten patients presented to us with pineal region meningioma. MRI was routinely used to define the tumor size, position, and its relevant complications while 3D-CTA was applied to define the blood supply of the tumor and the venous complex (VC) shift before operations. Most of the meningiomas had developed at both sides of the tentorial plane and extended laterally with typical characteristics of a pineal region tumor.

**Results:**

All tumors were completely removed surgically without any injury to the VC. Postoperative intracranial infection occurred in one case who recovered after antibiotics were given. Postoperative intraventricular hemorrhage and pneumocephalus were found in one case, but fully recovered after conservative treatment. In the nine cases of concurrent hydrocephalus, this was gradually relieved in eight patients and the single case that became aggravated was successfully treated with ventriculoperitoneal shunt. Moreover, the follow-up MRI examinations did not indicate any recurrence of the meningiomas.

**Conclusion:**

We found that the use of Poppen's approach is strongly supported for the successful removal of pineal region meningiomas without serious complications.

## Background

The pineal region contains certain tissues that have distinct histological characteristics, including the pineal gland and several parapineal structures such as the posterior third ventricle and the aqueduct, brain, dura and vessels [1]. The list of tumors that can arise within the pineal region is extensive and encompasses germ cell tumors, pineal parenchymal cell tumors, gliomas and meningiomas, in which meningiomas are a rare clinical occurrence, as they only account for about 8% of all pineal region tumors [2]. However, treatments for meningiomas within the pineal region using microsurgical manipulation is highly challenging as a result of several factors, such as its deep location, complicated surrounding vascular networks including the deep veins that drain midline brain structures and supplying arteries, and the anatomical obstacles of the tentorium and falx [3].

Recent reports of surgical interventions in the pineal region for meningiomas are mostly gained from independent studies that describe surgical approaches, such as Poppen's approach, Krause's approach and combinations of the two approaches. It can be very difficult for clinicians to choose the most appropriate treatment approach [2,4,5]. The ten meningiomas in this study were successfully removed surgically using Poppen's approach. Before each surgery, 3D-CTA examinations were performed to assist Poppen's approach. The findings of this study suggested that preoperative CTA can greatly aid in the understanding of the anatomical relationship between the deep venous system and the tumor and its blood supply. Additionally, this study demonstrates that Poppen's approach is clinically feasible for the treatment of meningiomas within the pineal region, and it provides a good prognosis for patients.

## Materials and methods

### Criteria of inclusion

(1). The sites of the meningiomas were restricted to the quadrigeminal cistern or the rear of the third ventricle. (2). The tumor originated in the dura at the origin of straight sinus or tentorial free edge and was mainly localized in the pineal region without severe adhesions. (3). Cases with tentorial meningiomas that protruded into the pineal region were excluded from the study [2].

### Clinical history

Four male and six female patients aged between 40-64 years (average age, 53.3 years) were recruited having suffered symptoms for a period of 3 days to 5 years. Among these patients, six cases were found to suffer from headaches, four cases had papilledema, three cases had hearing loss, one case exhibited limb hemiparesis, five cases displayed dizziness, four cases presented with ataxia and one case had paralysis of upgaze. Among the ten patients, six had a KPS score ≥80, indicating a satisfactory quality of life, while four had a KPS score <80 which indicated an unsatisfactory quality of life.

### Radiological examination

**Figure 1:**
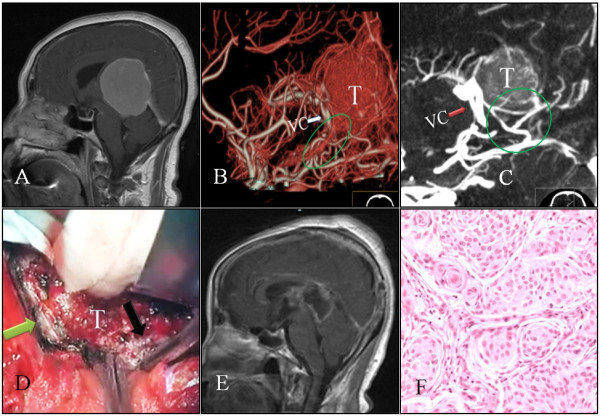
**Case 5: A: Sagittal sections of MRI scans showing a huge meningioma which was contrast enhanced in the pineal region**. It grows below and above the tentorium. B-C: CTA images show moderate tumor (T) staining. The meningioma is supplied by the posterior branch of the cerebral artery (ellipse). VC shows downward shifting (arrow). D: An image of the opening of the cerebral falx (black arrow) and tentorium (green arrow) that exposed the tumor (T), in order to carry out the piecemeal excision. E: Postoperative contrast-enhanced sagittal MRI sections showing the surgical removal of the pineal region meningioma; the artefact of the early postoperative period can be seen. F: Histopathological section of the tumor showing an endothelial WHO grade I meningioma (HE ×200).

**Figure 2:**
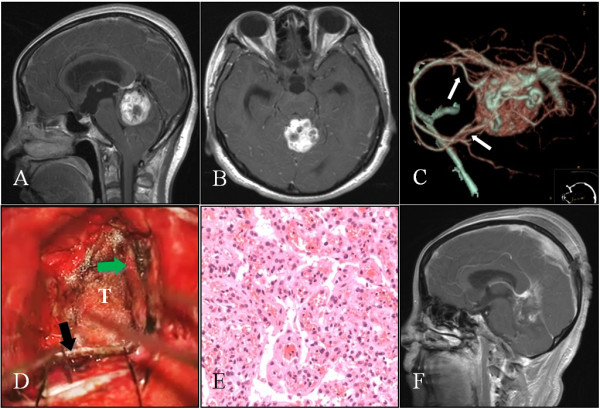
**Case 8: A-B: Axial and sagittal sections of MRI scans showing an enhancing contrast tumor in the pineal region, below the tentorium**. C: CTA image showing intense tumor staining. The tumor is supplied by the posterior cerebral and superior cerebellar arteries (arrow). D: Exposure of the tumor (T) after sectioning of the tentorium (green arrow), and cerebral falx (black arrow). E: Histopathological section of the tumor showing a vascular WHO grade I meningioma (HE, x200). F: Postoperative contrast-enhanced sagittal section of the MRI showing no residual tumor.

**Figure 3:**
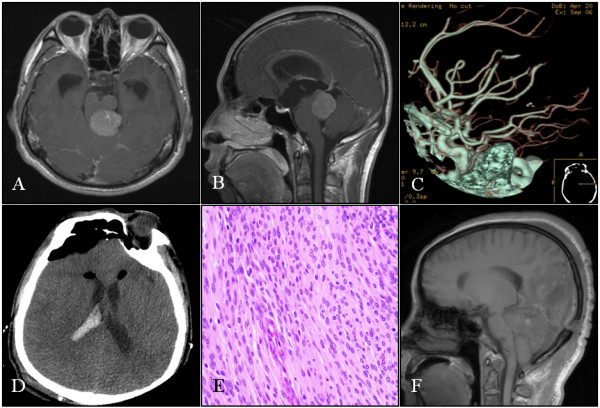
**Case 10: A-B: Axial and sagittal sections of MRI scans that show a contrast-enhanced tumor in the pineal region below the tentorium compressing the brainstem**. C: CTA did not demonstrate tumor staining or a feeding artery. D: Postoperative CT scan showing hemorrhage in the lateral ventricle and pneumocephalus. E: Histopathological section of the tumor showing a WHO grade II meningioma (HE, x200). F: Postoperative sagittal MRI section showing no residual tumor.

(1). Magnetic resonance imaging (MRI) was used for defining the parameters as follows [6,7]. The size of meningiomas ranged from 1.8 cm to 5.3 cm (average, 3.45 cm) in diameter and the relationship between the tentorium cerebelli and tumor was divided into infratentorial > supratentorial in three cases (Figure 1), supratentorial > infratentorial in two cases, supratentorial = infratentorial in three cases and infratentorial in two cases (Figure 2 and Figure 3). Additionally, hydrocephalus was found in a mild form in five cases, moderate in one case, severe in three patients and no hydrocephalus was present in one case.

(2). Computed tomography angiography (CTA) was applied to detect tumor staining, venous complex (VC, which includes the medial occipital vein, basal vein, internal cerebral veins and great cerebral vein [8]) shift and blood supply [9]. The degrees of staining were severe in four cases, moderate in two cases, mild in three cases and no staining in one case. The VC shifted downwards in three cases, upwards in one case, laterally in four cases and no shift was observed in two cases. The blood supply to the tumor was from the pericallosal arterial branches in one case, the posterior choroidal artery branches in two cases, the posterior cerebral artery branch in two cases, the posterior cerebral and superior cerebellar artery branches in one case, the dura of the origin of straight sinus in three cases and was unspecified in one case.

### Surgical procedures

After the induction of general anesthesia, lumbar drainage was performed and the three-way tap was kept in the closed position. Patients were positioned laterally and a bone window with a size of approximately 9 × 7 cm was opened on the side of the tumor to expose the superior sagittal sinus, torcular herophili and transverse sinus. After releasing some of the cerebrospinal fluid via the lumbar puncture drainage, the dura was opened, and the occipital lobe was lifted obliquely from the junction of the lower and median edges after reaching the angle of the sagittal sinus and the tentorium cerebelli. The bridging vein from the occipital lobe into the sagittal sinus was ligated and transected. Following the tentorium cerebelli and the cerebral falx leading to the pineal region, the tentorium cerebelli was transected parallel to the straight sinus in order to expose the tumor. If necessary, the cerebral falx was incised to help expose the tumor. The VC was well protected if found to be associated with the tumor, or it was found after the removal of the tumor. A Cavitron ultrasonic surgical aspirator (CUSA) was used for the intracapsular excision of the tumor, while protecting the medial occipital vein [10].

### Postoperative care

All patients were monitored closely for any complications and resolution of any hydrocephalus. For patients with WHO grade II atypical meningiomas, adjuvant radiotherapy was given after discharge. Patients with preoperative hydrocephalus were examined every month with CT after discharge; the single case without preoperative hydrocephalus was followed-up with monthly telephone conversations. MRI examination was performed during the follow-up period to check for any recurrence of meningiomas.

### Statistical methods

Using SPSS 13.0 software, the preoperative and postoperative KPS score were analyzed with the X^2 ^test and *P*-values < 0.05 were considered to be statistically significant. The data were shown in table 1.

**Table 1:** Clinical histories and previous observations are summarized

**Case No**.	Sex/age	Duration	Clinical presentation	KPS	CTA	Blood supply	Size (cm)	Position	Hydrocephalus
1	Female/46 years	8 months	Headache, Papilledema, Hearing loss	80	Severe staining, VC shift upward	Pericallosal arterial branches	4.7 × 2.1 × 3.0 (an average of 3.3)	Infratentorial > supratentorial	Moderate

2	Male/61 years	2 months	Headache, papiledema, limb hemiparesis	70	Moderate staining, VC shift left	Posterior choroidal artery branches	4 × 4 × 3 (an average of 3.7)	Supratentorial = infratentorial	Severe

3	Male/42 years	3 months	Headache, dizziness, papilledema, Ataxia	70	Mild staining, VC shift left	dura of the origin of straight sinus	3.0 × 2.5 × 2.0 (an average of 2.5)	Infratentorial > supratentorial	Severe

4	Female/64 years	4 years	Dizziness, Ataxia	90	Mild staining, VC shift left	dura of the origin of straight sinus	4.3 × 4.1 × 3.3 (an average of 3.9)	Supratentorial > infratentorial	Mild

5	Female/64 years	5 years	Papilledema, memory loss, ataxia, paralysis of upgaze	60	Severe staining, VC shift downward	posterior cerebral artery branch	5.0 × 5.0 × 4.0 (an average of 4.7)	Infratentorial > supratentorial	Mild

6	Female/62 years	3 days	Headache	90	Mild staining, VC shift downward	dura of the origin of straight sinus	4.6 × 3.9 × 4.2 (an average of 4.2)	Supratentorial > infratentorial	Mild

7	Male/41 years	2 years	Headache	90	Moderate staining, VC shift right	posterial choroidal artery branches	4 × 3 × 3 (an average of 3.3)	Supratentorial = infratentorial	None

8	Female/60 years	4 years	Dizziness, hearing loss	80	Severe staining, VC Shift downwards	posterior cerebral artery branch	4.7 × 5.2 × 6.0 (an average of 5. 3)	Supratentorial = infratentorial	Mild

9	Female/53 years	3 years	Dizziness, ataxia	80	Severe staining, VC unaffected	posterior cerebral and superior cerebellar artery branches	2 × 2 × 1.5 (an average of 1.8)	Infratentorial	Mild

10	Male/40 years	2 years	Headache, dizziness, hearing loss	70	No staining and VC shift	not specified	2 × 2 × 1.5 (an average of 1.8)	Infratentorial	Severe

## Results

### Intraoperative results

Out of the ten cases whose meningiomas were removed, six cases originated from the pineal region and four cases originated from the dura of the origin of straight sinus. In eight cases, the VC was retained clearly after tumor resection. In one case, the VC was retained but the basal vein on that side was absent. In another case, the VC was retained, but the bridging veins of the superior cerebellar vermis were ligated and transected.

### Postoperative results

The pathological findings revealed eight patients with WHO grade I, and two had WHO grade II meningiomas (patients were given conventional radiotherapy postoperatively). One patient developed a postoperative intracranial infection and recovered after antibiotics were administered. Postoperative intraventricular hemorrhage and pneumocephalus occurred in one case, but this patient recovered and was discharged after conservative treatment. The remaining eight patients had no postoperative complications.

### Follow-up observations

All patients were followed-up for a period of 6-24 months (average, 14 months). Preoperative concurrent hydrocephalus in nine patients improved after the operation, and eight of them remained under review afterwards. However, there was an aggravation of the hydrocephalus in one of the nine patients and this patient was treated with a ventriculoperitoneal shunt. The MRI scans that were performed in the follow-up period showed no recurrence of any of the meningiomas. During follow-up, preoperative symptoms improved to varying degrees; KPS ≥80 was found in nine patients and KPS <80 was only found in one case, which was significantly different compared with the preoperative score (X^2 ^= 2.4, *P *< 0.05). The data were shown in table 2.

**Table 2:** Surgical treatments and the follow-up record for each patient case are summarized

	Intraoperative		Postoperative			Follow-up			
	
**Case No**.	Origin	VC	Type	Complication	Other therapy	Time	KPS	Hydrocephalus	Relapse
1	Pineal region	Retained and clear	Meningioma, WHO grade I	No	No	6 months	90	Mild	No

2	Pineal region	Retained and clear	Nontypical meningioma, WHO grade II	No	Conventional Radiation	12 months	70	Shunt	No

3	Dura of the origin of straight sinus	Retained and clear	Meningioma, WHO grade I	No	No	18 months	90	Mild	No

4	Dura of the origin of straight sinus	Bridging vein ligation	Meningioma, WHO grade I	No	No	12 months	100	No	No

5	Pineal region	Retained and clear	Meningioma, WHO grade I	No	No	24 months	80	No	No

6	Dura of the origin of the straight sinus	Retained and clear	Meningioma, WHO grade I	Intracranial infection	No	12 months	100	No	No

7	Pineal region	Retained and basal vein absent	Meningioma, WHO grade I	No	No	18 months	100	No	No

8	Dura of the origin of the straight sinus	Retained and clear	Meningioma, WHO grade I	No	No	12 months	100	No	No

9	Pineal region	Retained and clear	Vascular meningioma, WHO grade I	No	No	12 months	100	No	No

10	Pineal region	Retained and clear	Nontypical meningioma, WHO grade II	Postoperative intraventricular hemorrhage and pneumocephalus	Conventional Radiation	24 months	90	Mild	No

## Discussion

The meningioma in the pineal region accounts are uncommon, but because of the benign biological behavior of meningiomas in the pineal region and its good prognosis, it is still worthy of study in order to define a standard surgical approach that will benefit patients [2,11,12]. When a pineal region meningioma grows bilaterally to the tentorial plane and extends laterally, the large variation of the tentorial angle will result in a complicated anatomical relationship between the tumor, the tentorium and the falx [13].

Currently, the infratentorial supracerebellar (Krause's) approach, occipital transtentorial (Poppen's) approach and the combination of both approaches are commonly used. Krause's approach is essentially a midline posterior approach to the pineal region. Its main advantage is that this approach located underneath the major deep veins, which lessens the chance of severe neurovascular compromise [3]. However, because of the presence of the tentorium, which produces restricted visualization at both the lateral and superior corners, Krause's approach provides a narrow operative field, which is a limiting factor. For pineal region tumors, the alternative was Poppen's approach, which provides an extensive and clearer view of the entire pineal region from above the tentorium [14]. Therefore, the choice of surgical approach depends on the relationship between the tumor and tentorium. If the tumor is located below the level of tentorium, Krause's approach should be used; otherwise, Poppen's approach is preferred. Moreover, if the tumor is too large to be removed using a single approach, then the combined approach should be performed [10,15,16].

However, in our department, we usually adopt Poppen's approach to divide the tentorium and falx to achieve adequate space during the operation. If the tumor was too large to be viewed completely in this approach, it is still feasible to lower the pressure inside the meningioma sac and pull the unrevealed part of tumor into the operative field before proceeding with the operation. The current ten cases mentioned in this report were all treated successfully and efficiently using this treatment approach.

Except for the falx and tentorium, the surgical removal of pineal region meningioma is also affected by the VC of the pineal region [17,18]. Pineal region meningioma commonly results in a shift of the surrounding structures of the quadrigeminal cistern, stretches the VC and disrupts the normal anatomical relationship among these structures, causing surgical difficulties [19]. Therefore, it is crucial to remove the tumor without damaging the VC, which makes the preoperative assessment of the relationship between the tumor and VC very important. Currently, CTA is a convenient technique that provides a three-dimensional visual reconstruction of the tumor and its blood supply. It combines the use of x-ray beams that are passed through the area of interest from various different angles to obtain projection images, which are then computerized into a three-dimensional image. CTA clearly shows the relationship between the VC and tumor and also protects the VC from damage during the operation.

In this study, all ten cases with pineal region meningiomas underwent preoperative CTA examination, which provided data regarding the feeding vessels to the tumor, tumor staining and VC shift. These results are consistent with the surgical findings during the operation, which demonstrated that CTA is a valuable tool to analyze tumor blood supply and VC shift preoperatively. It is a useful technique to detect the feeding vessels of the tumor during tumor removal and to reduce both Intraoperative blood loss and operative time. The pineal region meningiomas can be divided into primary and secondary meningiomas [2,11,19]. Primary meningiomas are derived from the connective tissue of the pineal gland or the velum interpositum. It is usually not adherent to the dura [20], whereas the secondary tumors originate from the dura of the origin of the straight sinus or the tentorial free edge and grows into the pineal region [21]. In this study retrospective analysis of CTA images of these pineal region meningiomas, combined with intraoperative findings, revealed that the extent of the tumor staining and tumor blood supply was related to tumor classification. Primary meningiomas had a clear arterial supply, which was mainly from the carotid arterial system. Therefore, CTA revealed moderate or high staining. Secondary meningiomas were found to have no specific feeding arteries and showed a weak staining pattern. This may be due to the poor blood supply that originates mainly from the meningeal branches of the external carotid system. In order to understand the structural relationship between tumor and other vessels fully, surgeons are advised to use 3D reconstructions preoperatively to avoid being misled by the radiological reports.

Konovalov has reported 10 cases of surgical treatment of pineal region meningiomas. These reported cases were all successfully treated using Poppen's approach with the patients placed in a semi-reclining position. In their cohort, six patients with occlusion of the great cerebral vein or branches of the VC all had a good prognosis [2]. In our study, the ten cases had similar clinical presentations and imaging features. However, in order to avoid the risk of air embolism, we adopted the lateral position for surgical treatment [22]. In addition, the VC and its tributaries were well-protected intraoperatively, and this was supported by the preoperative CTA examination and the technological advancement of microsurgical techniques. Moreover, Lozier systematically reviewed pineal region meningiomas that originated from the velum interpositum, and used Krause's approach for treatment, achieving a good prognosis [23]. Ziyal reported a combined approach to provide a wider exposure of the pineal region with less brain retraction compared to Poppen's or Krause's approach alone. This alternative treatment is applicable to certain large and giant tumors of the pineal area. As demonstrated in their study, the combined approach was successful in the removal of large pineal region tumors in six cases, including four tentorial meningiomas, one pineocytoma, and one epidermoid cyst [4].

We prefer Poppen's approach as it provides the shortest distance to reach the tumor and the widest panoramic view for removing the tumor. Although Poppen's approach can easily damage midline structures, such as the VC and visual cortex, because it stretches the occipital lobe, this is preventable. Methods of prevention are as follows: 1) open the cerebral falx and tentorium to expose the midline and bilateral structures; 2) protect the VC with the use of CUSA or pushing VC in other directions than just superiorly; and 3) place a preoperative indwelling lumbar drain to reduce intracranial pressure and retract the occipital lobe to reduce occipital visual cortex damage. When there is obstructive hydrocephalus, the most common form of obstruction is not complete, which means that some CSF can be released intraoperatively. Even when the obstruction is complete, some CSF can leak through the subarachnoid space. Sometimes, a small volume of CSF can relieve the degree of retraction injury to the occipital lobe. Two cases in our study (cases 9 and 10, respectively), which were located entirely below the tentorium, fitted the criteria Krause's approach according to the relevant principles of the treatment of pineal region meningioma. However, we used Poppen's approach and opened the tentorium so that the tumor and its feeding arteries were fully exposed, which showed immense practicability.

In addition to protecting the VC, it is also very important to treat any hydrocephalus, which is the key factor in influencing the prognosis. Pineal region tumors affect the rear of the third ventricle and cerebral aqueduct, resulting in obstructive hydrocephalus. Previously, it was reported that producing a surgical window on the rear of the third ventricle facilitates the circulation of cerebrospinal fluid; however, this might lead to more serious injury [24]. In our case studies, hydrocephalus was relieved or disappeared in eight of our patients after the removal of tumors. In one case, hydrocephalus was aggravated but was successfully treated with a cerebral shunt.

## Conclusion

In summary, for meningiomas in the pineal region, 3D-CTA is of great clinical value to distinguish the anatomical relationship between the tumor, peripheral arteries and venous complex. This case series strongly supports the use of Poppen's approach in the surgical treatment of pineal region meningiomas.

## Competing interests

The authors declare that they have no competing interests.

## Authors' contributions

LYQ wrote the initial draft. LYQ, ZG and WHL contributed equally to this work. YJL is the operator and guarantor. All authors read and approved the final manuscript.
